# Interleukin-12p70 Expression by Dendritic Cells of HIV-1-Infected Patients Fails to Stimulate *gag*-Specific Immune Responses

**DOI:** 10.1155/2012/184979

**Published:** 2012-07-12

**Authors:** Ellen Van Gulck, Nathalie Cools, Derek Atkinson, Lotte Bracke, Katleen Vereecken, Marc Vekemans, Viggo F. I. Van Tendeloo, Zwi N. Berneman, Guido Vanham

**Affiliations:** ^1^Virology Unit, Department of Microbiology, Institute of Tropical Medicine (ITMA), 2000 Antwerp, Belgium; ^2^Laboratory of Experimental Hematology, Vaccine and Infectious Disease Institute (Vaxinfectio), Faculty of Medicine and Health Sciences, University of Antwerp, 2610 Wilrijk, Belgium; ^3^Unit of HIV/STD, Department of Clinical Sciences, ITMA, 2000 Antwerp, Belgium; ^4^Center for Cell Therapy and Regenerative Medicine, Antwerp University Hospital (UZA), Wilrijkstraat 10, 2650 Edegem, Belgium; ^5^Department of Biomedical Sciences, Faculty of Pharmaceutical, Veterinary and Biomedical Sciences, University of Antwerp and Faculty of Medicine and Pharmacy, Free University of Brussels, Belgium

## Abstract

A variety of immune-based therapies has been developed in order to boost or induce protective CD8^+^ T cell responses in order to control HIV replication. Since dendritic cells (DCs) are professional antigen-presenting cells (APCs) with the unique capability to stimulate naïve T cells into effector T cells, their use for the induction of HIV-specific immune responses has been studied intensively. In the present study we investigated whether modulation of the activation state of DCs electroporated with consensus codon-optimized HxB2 *gag* mRNA enhances their capacity to induce HIV *gag*-specific T cell responses. To this end, mature DCs were (i) co-electroporated with mRNA encoding interleukin (IL)-12p70 mRNA, or (ii) activated with a cytokine cocktail consisting of R848 and interferon (IFN)-**γ**. Our results confirm the ability of HxB2 *gag*-expressing DCs to expand functional HIV-specific CD8^+^ T cells. However, although most of the patients had detectable *gag*-specific CD8^+^ T cell responses, no significant differences in the level of expansion of functional CD8^+^ T cells could be demonstrated when comparing conventional or immune-modulated DCs expressing IL-12p70. This result which goes against expectation may lead to a re-evaluation of the need for IL-12 expression by DCs in order to improve T-cell responses in HIV-1-infected individuals.

## 1. Introduction

Accumulating evidence suggests that human immunodeficiency virus (HIV)-specific T cells play a critical role in controlling viral replication in chronic HIV infection [1–5]. Although most individuals show detectable levels of HIV-specific CD8^+^ T cell responses [6], a defect of HIV-specific CD8^+^ T cells to expand *in vitro* has been previously reported [7]. Evaluation of *ex vivo* CD8^+^ T cells further indicates qualitative defects, such as low avidity, limited polyfunctionality, and T cell exhaustion which could explain why, despite a clear-cut cellular immune response, most HIV-infected patients show clinical progression in the absence of antiretroviral therapy. Even under highly active antiretroviral therapy (HAART), the functional capacity of HIV-specific CD8^+^ T cells, that is, secretion of soluble mediators and ability to expand, is not restored [8, 9]. Therefore, a major goal of many candidate HIV therapeutic vaccines in development is to stimulate the cellular arm of the immune system, in particular HIV-specific CD4^+^ and CD8^+^ T cells [10]. Unfortunately, these new therapies and vaccines do not deliver the expected success yet. For instance, Autran et al. reported significantly elevated viral loads in chronically infected patients compared to the placebo group after discontinuation of therapy following immunization with vCP1452, the HIV-recombinant canarypox vaccine (ALVAC-HIV) [11]. Remarkably, patients receiving more doses of the vaccine required treatment resumption more rapidly. Analysis of these results strongly suggested that the vaccine failed to elicit protective HIV-specific CD8^+^ T cell responses [12]. Moreover, mainly induction of activated CD4^+^ T cell responses was demonstrated in vaccines, which increased target cell availability and rendered patients potentially more susceptible to disease progression. This observation underlined the importance of potent CD8^+^ T cell responses for viral control and strongly suggested that, in order to provide a more effective strategy to induce viral control, it is important that therapeutic vaccine strategies induce robust and broad CD8^+^ T cell responses, while only modestly activating CD4^+^ T cell responses.

Since dendritic cells (DCs) are professional antigen-presenting cells (APCs) with the unique capability to stimulate naïve T cells into effector T cells [13, 14], their use for the induction of HIV-specific immune responses has been studied intensively. Some of the key factors that determine the ability of DCs to improve the quality and quantity of T cell responses include the antigen-loading strategy, the expression levels of costimulatory molecules and cytokines, as well as signals for activation of DCs [15]. We [16–18] and others [19–25] have previously shown that DCs transfected with mRNA-encoding antigens are superior to other loading strategies for induction of immune responses. Additionally, a multitude of products has been used for activation of DCs, including members of the tumor necrosis factor (TNF) family, Toll-like receptor (TLR) ligands, and type I and II interferons; other activators include various cytokines, chemokines, and prostaglandins. The current golden standard method to induce activation of DCs for clinical application is based on a cocktail of proinflammatory cytokines (IL-1*β*, TNF-*α* and IL-6) and prostaglandin (PG) E_2_ [26]. It has, however, been demonstrated earlier that a simplified cocktail consisting of only TNF-*α* and PGE_2_ generates a similar mature HLA-DR^+^CD86^+^CD80^+^CD83^+^CCR7^+^ DC phenotype [27, 28]. While PGE_2_ increases the expression of CCR7 and hence the capacity of DCs to migrate to the regional lymph nodes through chemotaxis by CCL-19 and/or -21 [29], PGE_2_ also inhibits interleukin (IL)-12p70 secretion by DCs, which is otherwise necessary for the development of an effective Th1 immune response [30, 31]. In order to increase IL-12p70 secretion, DCs can be electroporated with mRNA encoding IL-12p70 [32]. However, too much IL-12 production can paradoxically contribute to T cell exhaustion [33, 34]. Since this should be avoided, conditions that induce endogenous production of IL-12p70 by DCs could be used [35]. Indeed, it was reported that DCs matured with type II interferon (IFN), that is, IFN-*γ*, together with IL-1*β* and TNF-*α* overcome such maturation-associated exhaustion [36, 37].

We previously demonstrated that DCs (from HAART-treated individuals), electroporated with consensus codon-optimized HxB-2 *gag* mRNA or autologous proviral-derived *gag* mRNA, efficiently expand HIV-specific T cells that secrete IFN-*γ*, IL-2, perforin, and other cytokines [16, 17]. In the present study we aimed at enhancing the immunogenic potential of DCs by varying the signals for activation of DCs and analyzing the effect of those activated *gag*-expressing DCs on various aspects of *gag*-specific CD8^+^ T cell responses, such as the ability to produce soluble mediators and to expand after restimulation with *gag*-derived peptides.

A variety of immune-based therapies have been developed in order to boost or induce protective CD8^+^ T cell responses in order to control HIV replication. Since dendritic cells (DCs) are professional antigen-presenting cells (APCs) with the unique capability to stimulate naïve T cells into effector T cells, their use for the induction of HIV-specific immune responses has been studied intensively. In the present study we investigated whether modulation of the activation state of DCs electroporated with consensus codon-optimized HxB2 *gag* mRNA enhances their capacity to induce HIV *gag*-specific T cell responses. To this end, mature DCs were (i) co-electroporated with mRNA encoding interleukin (IL)-12p70 mRNA, or (ii) activated with a cytokine cocktail consisting of R848 and interferon (IFN)-*γ*. Our results confirm the ability of HxB2 *gag*-expressing DCs to expand functional HIV-specific CD8^+^ T cells. However, although most of the patients had detectable *gag*-specific CD8^+^ T cell responses, no significant differences in the level of expansion of functional CD8^+^ T cells could be demonstrated when comparing conventional or immune-modulated DCs expressing IL-12p70. This result which goes against expectation may lead to a re-evaluation of the need for IL-12 expression by DCs in order to improve T-cell responses in HIV-1-infected individuals.

## 2. Materials and Methods

### 2.1. Study Population

Peripheral blood samples (100 mL) from HAART-treated HIV-1-seropositive individuals were recruited at the clinical department of the Institute of Tropical Medicine of Antwerp according to institutional guidelines and after obtaining informed consent. Patients included had undetectable viral load levels and a CD4^+^ T cell count above 300 cells/*μ*L. The study was approved by the Institute's Ethics Committee and followed the tenets of the Declaration of Helsinki.

### 2.2. Plasmid DNA Constructs

The pGEM4Z/h*gag*/A64 (pGEMh*gag*) plasmid was kindly provided by Professor Kris Thielemans (Laboratory of Physiology and Immunology, Free University of Brussels) and was used to prepare a humanized (codon-optimized) mRNA encoding the HxB-2 HIV-1 *gag* protein (hHxB-2 *gag*) [38]. The pGEM4Z/hIL-12/A64 plasmid was kindly provided by Professor Erik Hooijberg (Medical Center of the Vrije Universiteit, Amsterdam, The Netherlands) and was used to prepare mRNA encoding IL-12p70 [32]. These plasmids were propagated in E. coli supercompetent cells (Stratagene, La Jolla, CA, USA) and purified on endotoxin-free Qiagen-tip 100 columns (Westburg, Leusden, The Netherlands). Next, the plasmids were linearized with SpeI (MBI Fermentas, St. Leon-Rot, Germany), purified using a PCR purification kit (Qiagen), and used as a DNA template for *in vitro* transcription. *In vitro* transcribed 5′ capped mRNA was generated using T7 mMessage mMachine kit (Ambion, Austin, TX, USA). Purification of mRNA was performed by DNase I digestion followed by LiCl precipitation, according to manufacturer's instructions. RNA concentration was assayed by spectrophotometrical analysis and stored at −20°C in small aliquots.

### 2.3. Generation of DCs

Peripheral blood mononuclear cells (PBMCs) were isolated by Lymphoprep (Lucron, De Pinte, Belgium) gradient separation. Next, CD14^+^ monocytes were directly isolated by CD14^+^ immunomagnetic selection (CD14 Reagent, Miltenyi Biotec, Bergisch Gladbach, Germany) according to manufacturer's instructions and directly used for *in vitro* DC differentiation as described before [39] in medium containing 2.5% pooled human serum (PHS, PAA company, Austria). The CD14-depleted cell fraction, designated as peripheral blood lymphocytes (PBLs), was cryopreserved and stored at −80°C for later use in DC/T cell cocultures. On day 6, DCs were activated (i.e., converted to mature DCs (mDCs)) for 24 hours by adding a cocktail of proinflammatory cytokines consisting of 2.5 ng/mL TNF-*α* (Roche Molecular Biochemicals, Mannheim, Germany) and 10^−7^ M prostaglandin E_2_ (PGE_2_, Sigma, St Louis, MO USA). In parallel, DCs were simultaneously generated under the same conditions, but the “basic” cocktail was supplemented with the following factors: 25 ng/mL IFN-*γ* (Invitrogen, Merelbeke, Belgium) and 2 *μ*g/mL R848 (Alexis, Zandhoven, Belgium) or 1 *μ*g/10^6^ cells mRNA encoding IL-12p70 was coelectroporated with *gag* mRNA.

### 2.4. DC Electroporation

After incubation with the maturation cocktail for 24 hours, DCs were harvested and electroporated with mRNA encoding *gag* antigen and when indicated with mRNA encoding IL-12p70. Electroporation of *in vitro* transcribed mRNA was performed as described previously [18, 40]. After electroporation, cells were directly used in DC/T cell coculture experiments.

### 2.5. Cytokine Release Assay

Quantitative detection of the cytokine expression profile of DCs was determined using a Th1/Th2 multiplex fluorescent bead immunoassay (Bender MedSystems, Vienna, Austria), according to manufacturer's instructions.

### 2.6. DC-Mediated Stimulation of HIV *gag*-Specific T Cell Responses

To evaluate the capacity of DCs to expand *gag*-specific T cells, *gag* mRNA-electroporated mature DCs were cocultured with autologous PBLs (ratio 1 : 10) in RPMI 1640 medium supplemented with 2.5% PHS. After 7 days, PBLs were harvested and analyzed using a “restimulation” IFN-*γ* ELISpot assay. Furthermore, a fraction of the cells was stimulated for an additional 7 days with cryopreserved HIV-1 *gag* mRNA-electroporated autologous DCs [40]. At day 14, these cells were analyzed using an IFN-*γ* ELISpot assay. In parallel, unstimulated PBLs (day 0) and PBLs stimulated with mock-electroporated DCs (day 7 and day 14) were used as a control.

### 2.7. IFN-*γ* ELISpot Assay

For detection of IFN-*γ*-producing HIV *gag*-specific activated T cells, PBLs were incubated at a concentration of 2.5 × 10^5^ cells/well with *gag* peptide pool (2 *μ*g/mL, NIH, Maryland, USA) in anti-human IFN-*γ* antibody (Diaclone, Besancon, France) coated 96 well-plates (Millipore), for at least 16 hours and not more than 24 hours. For the detection of spots, a biotin-labelled anti-human IFN-*γ* antibody (Diaclone) was used. Frequencies of antigen-specific IFN-*γ*-secreting cells were calculated based on the number of spots counted using an automated iSpot Reader system (AID GmbH, Strassberg, Germany). For the definition of a positive response, guidelines from the Cancer Vaccine Consortium were followed: per 10^6^ PBLs, the mean antigen-specific spot count for a donor and condition must be greater than or equal to 50 spots and at least three times as high as the background reactivity [41, 42].

### 2.8. Statistics

The results are expressed as mean ± standard deviation. Comparisons were validated using Wilcoxon signed rank test and 2-way Anova using GraphPad version 5 software (Prism, La Jolla, CA, USA). A *P* value of ≤0.05 was considered as statistically significant.

## 3. Results

### 3.1. Do Immune-Modulated DCs Produce IL-12p70 and/or Other Cytokines?

**Figure 1:**
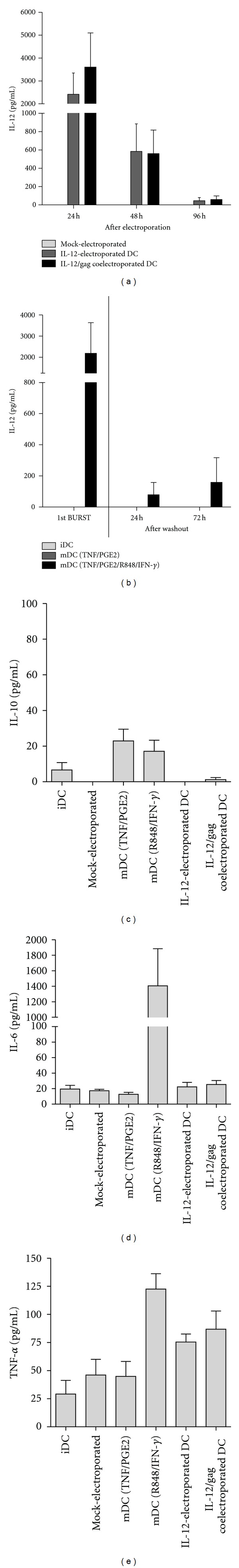
Production of pro- and anti-inflammatory cytokines by DCs modulated to express IL-12p70. (a) IL-12 secretion by DCs electroporated with IL-12p70-encoding mRNA. DCs were examined after incubation with a proinflammatory cytokine cocktail consisting of TNF-*α* and PGE_2_ and electroporated with mRNA encoding IL-12p70 or with IL-12p70-encoding mRNA in combination with hHxB-2 *gag*-encoding mRNA. As a control mock-electroporated mature DCs were examined. DC culture supernatant was collected 24 h, 48 h, and 96 h after electroporation. Results are shown as mean ± standard deviation (*n* = 5). (b) IL-12 secretion by immune-modulated DCs. DCs were examined after incubation with a proinflammatory cytokine cocktail consisting of TNF-*α* and PGE_2_ or activated under the same conditions supplemented with R848 and IFN-*γ*. As a control untreated, that is, immature DCs (iDCs) were examined. DC culture supernatant was collected 24 h after activation with proinflammatory stimuli, as well as 24 h and 72 h after a washout experiment. Results are shown as mean ± standard deviation (*n* = 5). (c), (d), and (e) Cytokine secretion by DCs activated under different experimental conditions and after a 24 h washout period. Values are shown for (c) IL-10, (d) IL-6, and (e) TNF-*α*. Results are shown as mean ± SD (*n* = 5).

We have previously described that mRNA electroporation is a highly efficient tool to induce transgene expression in monocyte-derived dendritic cells [16–18]. Indeed, electroporation of Mo-DCs with HxB-2 *gag* mRNA resulted in efficient expression of *gag* protein compared to mock-electroporated DCs (see Supplementary Figure 1(A) available online at doi: 10.1155/2012/184979). Here, DCs were activated using a cocktail of proinflammatory cytokines consisting of (i) TNF-*α* and PGE_2_ or (ii) TNF-*α*, PGE_2_, IFN-*γ*, and R848. Simultaneously, TNF-*α*/PGE_2_ matured DCs were coelectroporated with IL-12p70-encoding mRNA. Next, IL-12p70 expression was assessed by means of IL-12p70 fluorescent bead immunoassay, 24 hours after electroporation. We demonstrate efficient translation of mRNA into secreted IL-12p70 protein, at concentrations ranging between 1 and 8 ng/mL by both DCs electroporated with IL-12p70-encoding mRNA (Figure 1(a)) and DCs activated using a cocktail of proinflammatory cytokines supplemented with IFN-*γ* and R848 (Figure 1(b)). In contrast, untreated DCs (i.e., immature DCs (iDCs)), mock-electroporated DCs or DCs activated with TNF-*α* and PGE_2_ alone did not secrete IL-12p70 (Figures 1(a) and 1(b)). In addition, the culture supernatant was collected at different time points after activation of DCs. As evidenced in this washout experiment, we are able to detect IL-12p70 secretion up to 72 hours after activation of DCs, albeit at significantly reduced levels (Figures 1(a) and 1(b)). Noteworthy, coelectroporation of mRNA encoding IL-12p70 and hHxB-2 *gag* did not affect the level of transgene expression (Figure 1(a) and Supplementary Figure 1(B)). Furthermore, secretion of other pro- and anti-inflammatory cytokines, that is, IL-10, IL-6 and TNF-*α*, was evaluated after washout as well. Whereas DCs stimulated with a cocktail consisting of TNF-*α*, PGE_2_, IFN-*γ*, and R848 secreted significantly higher levels of IL-6 (Figure 1(d)), no differences could be detected in other investigated experimental conditions (Figures 1(c), 1(d) and 1(e)). 

### 3.2. Can IL-12 mRNA Electroporation of DCs Improve the Expansion of HIV *gag*-Specific T Cells?

**Figure 2:**
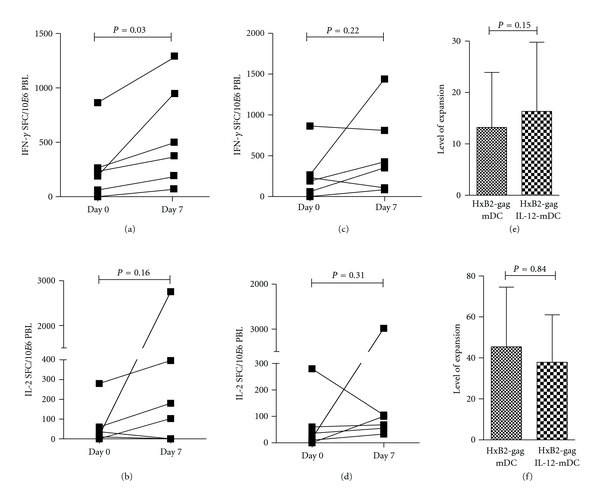
Effect of coelectroporation of DCs with *IL-12p70* mRNA on HIV *gag*-specific T cell responses after 1 week of stimulation. Freshly thawed PBLs were directly incubated in ELISpot with HIV *gag*-derived peptides (Day 0) or PBLs were stimulated for one week with autologous HIV *gag* mRNA-electroporated DCs. For this, DCs were either matured with a simplified cytokine cocktail (TNF-*α* and PGE_2_) (a) and (b) or DCs were matured with this simplified cytokine cocktail in combination with *IL-12p70* mRNA electroporation (c) and (d). Thereafter, cocultured PBLs were restimulated with HIV *gag*-derived peptides and evaluated in ELISpot for detection of antigen-specific IFN-*γ* (a), (c) and (e) or IL-2 (b), (d), and (f) secretion. Results are shown as mean of triplicate analyses for each donor displaying a positive response (*n* = 6), as defined in the Materials and Methods section.

Since IL-12 is a key cytokine supporting cytotoxic CD8^+^ T cell responses through the polarization of a Th1 immune response [30, 31], we investigated if hHxB-2 *gag* mRNA-electroporated DCs cotransfected with IL-12p70 mRNA can expand the number of cytokine-secreting HIV *gag*-specific T cells. To this end, DCs matured with TNF-*α* and PGE_2_ were electroporated with mRNA encoding HIV *gag* or with *IL-12p70* mRNA or with both mRNAs and subsequently cocultured with autologous PBLs for 1 week. Next, stimulated PBLs were restimulated with HIV *gag* peptide pool in an IFN-*γ* and/or IL-2 ELISpot assay. The induced immune responses were compared with the responses generated by PBLs stimulated with HIV *gag* peptide pool at day 0. As demonstrated in Figure 2, DCs electroporated with HIV *gag* mRNA induced expansion of HIV *gag*-specific T cells (Figures 2(a) and 2(b)). However, DCs cotransfected with *IL-12p70* and HIV *gag* mRNA did not enhance expansion of HIV *gag*-specific T cells (Figures 2(c) and 2(d)). An equally efficient expansion of HIV *gag*-specific T cells was observed after seven days, regardless of which DC maturation cocktail was used (Figures 2(e) and 2(f)). 

### 3.3. Can an IL-12-Inducing DCs Maturation Cocktail Enhance the Expansion of HIV *gag*-Specific T Cells?

**Figure 3:**
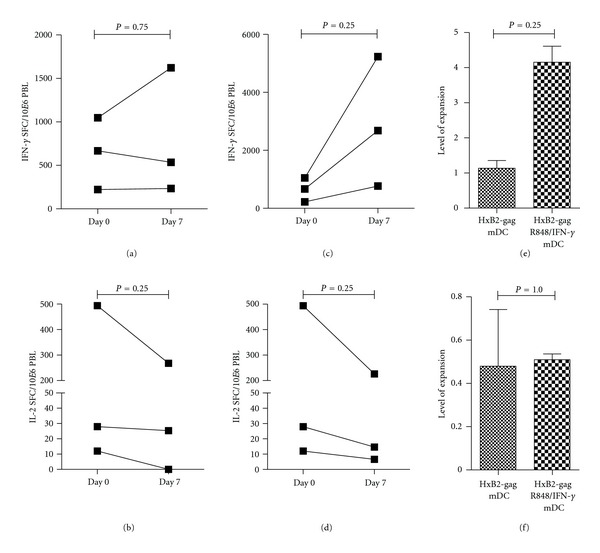
Effect of addition of TLR7/8 ligand, R848, and IFN-*γ* for activation of DCs on HIV *gag*-specific T cell responses after 1 week of stimulation. Freshly thawed PBLs were directly incubated in ELISpot with HIV *gag*-derived peptides (Day 0) or PBLs were stimulated for one week with autologous HIV *gag* mRNA-electroporated DCs. For this, DCs were either matured with a simplified cytokine cocktail (TNF-*α* and PGE_2_) (a) and (b) or DCs were matured with a simplified cytokine cocktail in combination with TLR7/8 ligand resiquimod (R848) and IFN-*γ* (c) and (d). Thereafter, cocultured PBLs were restimulated with HIV *gag*-derived peptides and evaluated in ELISpot for detection of antigen-specific IFN-*γ* (a), (c), and (e) or IL-2 (b, d, and f) secretion. Results are shown as mean of triplicate analyses for each donor displaying a positive response (*n* = 3), as defined in the Materials and Methods section.

Previously, it has been shown by others that stimulation of DCs with R848 together with IFN-*γ* can abrogate the PGE_2_-induced impairment of DCs to produce IL-12p70. In other words, DCs stimulated with a cocktail consisting of TNF-*α*, PGE_2_, IFN-*γ*, and R848 can secrete high amounts of IL-12p70 [36, 37]. In contrast to IL-12p70-cotransfected DCs, PBLs stimulated for one week with DCs matured with this “improved” cytokine cocktail produced more IFN-*γ* than PBLs stimulated with standard matured DCs, albeit not statistically significant (Figures 3(a), 3(c), and 3(e)). However, this improved maturation cocktail, consisting of TNF-*α*, PGE_2_, R848, and IFN-*γ*, did not enhance the expansion of HIV *gag*-specific IL-2-secreting T cells.

### 3.4. Can Immune-Modulated hHxB-2 *gag* mRNA-Electroporated DCs Induce Long-Lasting HIV *gag*-Specific Immune Responses?

**Figure 4:**
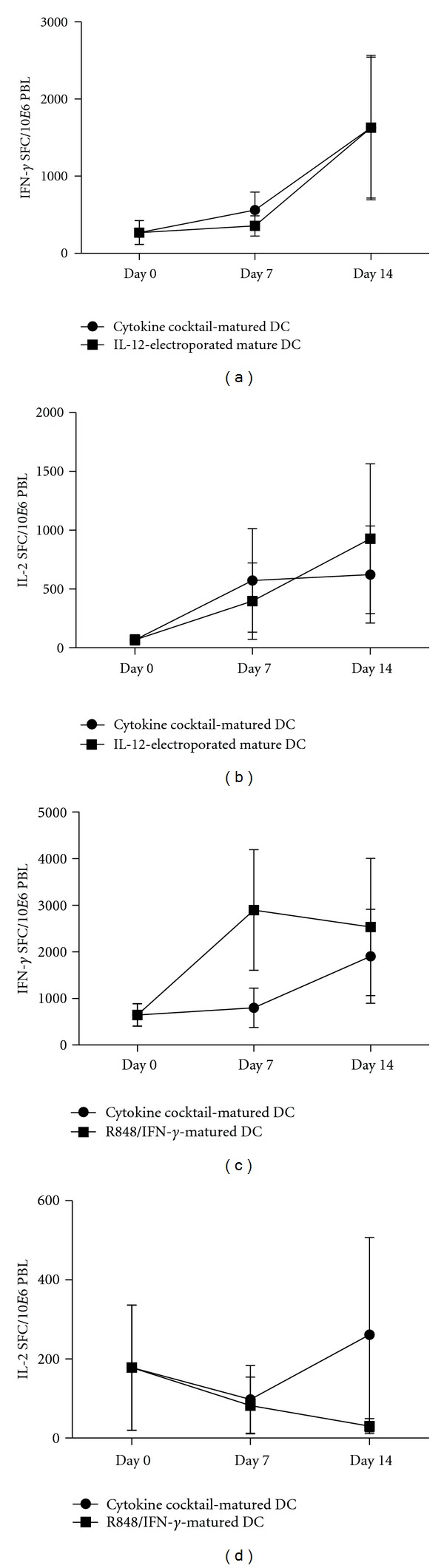
Stimulatory capacity of DCs from HIV-seropositive individuals after a second stimulation round. Freshly thawed PBLs were directly incubated in ELISpot with *gag*-derived peptides (Day 0), or PBLs were stimulated for one week with autologous *gag* mRNA-electroporated DCs (day 7), or PBLs were stimulated for a second week with thawed autologous electroporated DCs and reevaluated in ELISpot (day 14). For this, DCs were matured with a simplified cytokine cocktail (TNF-*α* and PGE_2_) or with a simplified cytokine cocktail in combination with *IL-12p70* mRNA electroporation (a) and (b), or with a simplified cytokine cocktail in combination with TLR7/8 ligand resiquimod (R848), and IFN-*γ* (c) and (d). Cocultured PBLs were evaluated for antigen-specific secretion of the following cytokines: IFN-*γ* (a) and (c) and IL-2 (b) and (d). Results are shown as mean of triplicate analyses for each donor displaying a positive response (*n* = 2), as defined in the Materials and Methods section.

Besides the need for potent CD4^+^ and CD8^+^ T cells, duration of the cellular response is another critical issue in the development of successful HIV vaccines. To evaluate this aspect, PBLs were stimulated for a second time with hHxB-2 *gag* mRNA-electroporated mature DCs at day 7. At day 14, stimulated PBLs were restimulated with HIV *gag* peptide pool in an ELISpot assay in order to detect antigen-specific IFN-*γ* and/or IL-2 secretion. While stimulation with DCs did not result in a decline of HIV *gag*-specific IFN-*γ*-producing T cells, no differences in the expansion of HIV *gag*-specific T cells could be observed after 14 days, regardless of which maturation cocktail was used (Figure 4).

## 4. Discussion

DC-based immunotherapy has been demonstrated to be safe with only minor local side effects reported in some clinical trials and has been shown to have clinical efficacy in HIV-infected patients [43–52]. Although an impressive amount of data has been obtained from the clinical trials completed thus far, no final conclusions can be drawn, because the design of the various clinical trials was different, few were really placebo-controlled and blinded, and all include only limited number of patients.

To mount an efficient immune response, at least three signals are necessary [13, 14, 53]. The first signal is the recognition of antigen peptide/major histocompatibility complex (MHC) conjugates presented by APCs. The second signal is provided through costimulatory molecules. The best-characterized costimulatory receptor-ligand interaction is that of CD28, expressed on naïve and memory T cells, with its ligands CD80 and CD86, expressed on APCs, such as DCs (reviewed in [54–56]). The third signal polarizes the effector T cell response toward a Th1, Th2, Th17, or Treg phenotype and is delivered by DCs through a number of soluble and membrane-bound ligands. One well-studied third signal agent is IL-12p70 for the induction of Th1 and cytotoxic T lymphocytes (CTLs) [57]. Until now, DCs matured with a proinflammatory cytokine-cocktail consisting of IL-1*β*, IL-6, TNF-*α*, and PGE_2_ have been the most commonly used in clinical settings [47, 52], although it is known that these DCs fail to produce IL-12p70 [58]. As an alternative, it was reported that PGE_2_-matured DCs cotransfected with mRNA encoding IL-12p70 and a tumor-associated antigen effectively secrete IL-12p70 and induce high avidity tumor-specific T cell responses *in vitro* [32].

Previously, we reported efficient *ex vivo* detection of HIV-1-specific immune responses in untreated HIV-1-seropositive persons [16], although they have a rather damaged immune system [59]. With the ultimate aim to develop an immunotherapy based on DCs, we subsequently provided proof of principle for the use of *gag* mRNA-electroporated monocyte-derived mature DCs as a possible strategy to improve T cell immunity under HAART [17, 60]. Consistent with our original hypothesis, the results presented here confirm the effective ability of *gag*-expressing DCs to expand functional HIV-specific CD8^+^ T cell responses. However, while it has been previously reported by others that IL-12p70-secreting DC preparations are always better for polarizing allogeneic CD4^+^ T cells and for sensitizing CD8^+^ T cells to tumor antigens compared to DCs secreting little or no IL-12p70 [30–32], we were unable to find a superior effect of IL-12p70-producing *gag* mRNA-electroporated DCs to induce and expand HIV-1 *gag*-specific CD8^+^ T cells. In doing so, we confirm the finding by Soares et al. that IL-12 is not mandatory for the Th1-inducing function of DCs [61]. Whether our observations are the results of an influence of HIV load on CTL responses cannot be ruled out and will need to be further investigated. However, we do not discount that other additional functions of antigen-specific CD8^+^ T cells that we have not analyzed, such as cytotoxicity and production of other soluble mediators, such as MIP-1*β* and TNF-*α*, could be influenced by stimulation with immune-modulated hHxB-2 *gag* mRNA-electroporated DCs. Indeed, others have shown that the quality of the CD8^+^ T cell response, that is, polyfunctional CD8^+^ T cells, is critically involved in viral suppression [62, 63], as it seems to be the case in long-term nonprogressors (LTNPs) and elite controllers (ECs) [64]. In addition to priming of T cells, the group of Banchereau has recently demonstrated that DCs may also directly signal naïve B cell differentiation through the production of IL-12p70 [65] and indirectly by promoting the differentiation of IL-21-producing T follicular helper (Tfh) cells [66, 67]. Although we were not able to demonstrate its critical role in cell-mediated immunity in HIV-infected patients in our experiments, these observations suggest that IL-12p70 could constitute a potent vaccine adjuvant in situations that may require both arms of the immune system, such as HIV [68]. Indeed, studies with rhesus macaques have concluded that IL-12p70 enhances the induction of specific antibody responses *in vivo* when used as vaccine adjuvant [69–71]. On the other hand, Traxlmayr et al. revealed a potential negative effect of peripheral blood *γδ* T cells on CD4^+^ and CD8^+^ immune responses in cancer patients undergoing immune therapy with IL-12p70-secreting DCs [72]. They suggest that IL-12-mediated triggering of *γδ* T cells may be part of a negative feedback mechanism for DC-controlled immune responses.

Previously, it has been shown by others that vaccines are only effective in models of persistent virus infection if they induce long-term immunological memory [73]. In support of this hypothesis, studies in macaques have shown that strong and early T cell responses can be generated by various vaccination regimens. However, none of these responses was long-lasting, as evidenced by a rapid decline of all generated beneficial immune responses [74–76]. We demonstrated here that stimulation of PBLs from HIV-infected patients with hHxB-2 *gag* mRNA-electroporated DCs maintained HIV-1 *gag*-specific IFN-*γ*- and IL-2-producing T cells in most instances.

In conclusion, while hHxB-2 *gag* mRNA-electroporated DCs expanded the number of HIV-1 *gag*-specific T cells, we were not able to further improve immune responses by immune modulation of DCs, that is, IL-12p70 mRNA cotransfection of DCs or activation of DCs with potential danger signals such as R848 and IFN-*γ*. Since we do not rule out effects on other arms of the immune system that may contribute to the control of HIV replication, we will further evaluate and refine this approach to develop consistent and long-lasting enhancement of immune responses with the ultimate aim of using them in therapeutic settings.
